# Using the health belief model to explain COVID-19 vaccination hesitancy in Dutch urban citizens under thirty

**DOI:** 10.1371/journal.pone.0279453

**Published:** 2023-01-26

**Authors:** Inge Merkelbach, Tessa Magnee, Shakib Sana, Jelena Kollmann, Paul Kocken, Semiha Denktas

**Affiliations:** Erasmus School of Social and Behavioural Sciences, Erasmus University Rotterdam, Rotterdam, Netherlands; Zagazig University Faculty of Human Medicine, EGYPT

## Abstract

**Background:**

Worldwide the Covid-19 pandemic resulted in drastic behavioral measures and lockdowns. Vaccination is widely regarded as the true and only global exit strategy; however, a high vaccination coverage is needed to contain the spread of the virus. Vaccination rates among young people are currently lacking. We therefore studied the experienced motivations and barriers regarding vaccination in young people with the use of the health belief model.

**Methods:**

We conducted a correlational study, based on a convenience sample. At the vaccination location, directly after vaccination, 194participants(16–30 years) who decided to get vaccinated at a pop-up location several weeks after receiving a formal invitation, filled out a questionnaire regarding their attitudes towards vaccination based on concepts defined in the health belief model. We used these concepts to predict vaccination hesitancy.

**Results:**

Younger participants and participants with lower educational levels report higher levels of hesitancy regarding vaccination (low education level = 38.9%, high education level = 25.4%). Perceived severity (M_hesitancy_ = .23, M_no hesitancy_ = .37) and susceptibility (M_hesitancy_ = .38, M_no hesitancy_ = .69) were not associated with hesitancy. Health related and idealistic benefits of vaccination were negatively associated with experienced hesitancy (M_hesitancy_ = .68, M_no hesitancy_ = -.37), while individualistic and practical benefits were not associated with hesitancy (M_hesitancy_ = -.09, M_no hesitancy_ = .05). Practical barriers were not associated with hesitancy (M_hesitancy_ = .05, M_no hesitancy_ = -.01), while fear related barriers were strongly associated with hesitancy (M_hesitancy_ = -.60, M_no hesitancy_ = .29).

**Conclusions:**

Health related, and idealistic beliefs are negatively associated with experienced hesitancy about vaccination, while fear related barriers is positively associated with experienced hesitancy. Future interventions should focus on these considerations, since they can facilitate or stand in the way of vaccination in young people who are doubting vaccination, while not principally opposed to it.

## Background

Worldwide the Covid-19 pandemic resulted in drastic behavioral measures and lockdowns. Mass vaccination is widely regarded as the only global exit strategy [1]. Mass vaccination seems a promising solution to prevent the implementation of new non-pharmaceutical interventions (NPIs) to contain the spread of the virus, such as strict behavioral measures. Mass vaccination has the potential to decrease the peaks in future waves; however, a critical (minimum) vaccination coverage is necessary to abolish or minimize NPIs [2].

At the start of the global vaccination campaign, the World Health Organization (WHO) estimated that a 70% vaccination coverage would be sufficient to reach herd immunity [3]. Due to the emergence of new and more contagious virus variants, the estimated needed vaccination coverage turned out to be an underestimation. The Delta variant for example, resulted in 50% to 100% more transmissions [4] as compared to the Alpha variant of the virus. The effects of the newly discovered Omicron variant are still unknown. Reaching a vaccination coverage as high as possible became thus even more urgent [5].

In order to prevent the spread of the Covid-19 virus, vaccination of just those who are at increased risk for experiencing severe symptoms (e.g., elderly or chronically ill groups [6]) is not sufficient. Reaching high vaccination rates in young people, who generally experience only mild symptoms after infection [7] as well, is crucial for containing the spread of the virus. This is especially the case because, while young people are seldomly admitted to hospitals or IC’s themselves, people younger than 30 years report to have more close contacts [8] than older people. Additionally, age is positively associated with compliance to Covid-19 measures [9]), making young people even more likely to contract and spread the virus, potentially causing outbreaks.

Unfortunately, willingness to get vaccinated is relatively low in young adults [10]. In the Netherlands, for example, this resulted in lower vaccinations rates among young adults. In 2021 less than 70% of people under thirty had been vaccinated [11]. Currently, among adults up to 24, this is still only 66.1%, and 70.3% for adults between 25 and 49 years of age as reported by the European Center of Disease Prevention and Control in October 2022. This percentage is high when compared to non-western countries (e.g., < 5% in 2021 in Saudi Arabia with almost half of the young population reporting to only take a vaccination when made mandatory [12]) but low as compared to neighboring countries such as Belgium (82.7% up to 25 years and 84.7% for people between 25 and 49, as reported by the European Center of Disease Prevention and Control in October 2022). Understanding in considerations of Dutch young people make when deciding about vaccination, is therefore crucial.

To be able to motivate and facilitate young people in their choice to get vaccinated against Covid-19, gaining insight in their decisions process is of critical importance. By understanding potential motivators and barriers for vaccination in this group, local authorities could design interventions targeted specifically at supporting relevant motivators, while lifting relevant barriers. According to the health belief model [13], an established social psychological health behavior change model developed to explain health-related behaviors such as use of health services, perceived susceptibility, and perceived severity, as well as perceived benefits and barriers explain if health behavior will occur (or not). In this line of reasoning, perceived susceptibility (i.e., estimated chances of contracting a condition) could be expected to be high in young groups due to high levels of social contact, while perceived severity (i.e., estimated chances of experiencing severe symptoms or consequences in case of contraction) of infection could be expected to be relatively low because of low hospitalization rates. However, other determinants of the model, such as how these attitudes are related to vaccine willingness remains unknown. Also, if and how perceived barriers and benefits affect vaccine willingness in young people remains to be studied. While for vulnerable subgroups protection of one’s own health might be considered an important benefit, for younger people benefits related to regained freedoms like travel and visiting hospitality and entertainment venues might carry more weight. Perceived barriers could also be distinctly different between age groups. Older groups might for example experience more accessibility related barriers, for example when travelling to vaccination locations, or when making online appointments which might be harder due to lower levels of digital literacy in this group [14]. On the other hand, younger age groups could experience higher levels of anxiety regarding side-effects (e.g., side effects regarding infertility and pregnancy related concerns [15], or higher levels of needle anxiety and -phobia which are more common among adolescents and young adults [16].

The aim of this study is to investigate if and how perceived susceptibility and severity, experienced benefits, and experienced barriers are related to experienced hesitancy regarding vaccination in an initially hard to reach group of young people (i.e., people who decided to get vaccinated at an easily accessible pop-up location without making an appointment, several weeks after receiving their formal invitation for vaccination).

## Methods

### Design

We used a cross-sectional survey study to gain insight in which predictors as defined in the Health Belief Model are associated with experienced hesitancy regarding vaccination. We decided to use a survey instead of interviews because the target group was expected to largely possess enough literacy skills to read, understand, and fill out a simple questionnaire. This study is part of the GP-COVERAGE (General Practice Covid study of Effects of Remote care in Areas with General practices with vulnerable patients) project, which was reviewed and approved by the Ethics Review Committee of the Erasmus University Rotterdam (#20–042). Because factors related to hesitancy regarding COVID-19 vaccination in this young age group was a new research topic we refrained from formulating specific hypotheses.

### Procedure

#### Context

The current study took place at an easy to reach (e.g., 2-minute walk from subway) pop-up vaccination location in Rotterdam, the Netherlands. During a 10-day period, at the end of July and beginning of August, vaccination was possible between 10:30 am and 8:30 pm without appointment. In the Netherlands, young people (born between 1991 and 2005) were invited for vaccination by the Dutch public health service between the 15^th^ of June and the 4^th^ of July. People who decided to get vaccinated at the current pop-up location had thus had at least three weeks’ time to make an appointment (via internet and phone), but instead decided to visit this location without an appointment. People could choose between a mRNA vaccine, or a vector vaccine. While the vaccination location was open to everyone, the municipality tried to mainly publicize this possibility among young people, for example by promotion through the digital networks of student associations.

#### Data collection

Data collection took place during four consecutive days of the 10-day period. Everyone who was willing to participate, was between 16 and 30 years of age, and able to answer a questionnaire in Dutch or English, was eligible for participation. After they had received a vaccination people were instructed to wait for at least 15 minutes (30 minutes for indicated subgroups such as those who had previously experienced side-effects in reaction to vaccination) at the vaccination location, according to a nationally implemented mandatory, medical observation period. At the start of this waiting period, participants were approached by a research assistant who asked if they were willing to participate and who explained the contents of the consent form such voluntariness (e.g., the freedom to stop participation at any time) and anonymity of participation (i.e., confidentiality), as well as data storage and handling. If participants agreed to fill out the survey, they received a consent form and survey as well as a pencil. All participants had to give written consent before starting the questionnaire. Participants filled out the questionnaires by themselves, but research assistants emphasized that participants could always ask for clarification or explanations if needed. Filling out the questionnaire took approximately 10 minutes. Pencils could be kept after filling out the survey, as a sign of appreciation and to prevent transmission of the virus between participants. Participants turned in the questionnaire when leaving the vaccination location, upon which research assistants checked if the questionnaire was fully answered and if participants had understood all questions. Participants did not receive any further reimbursements.

### Materials

We designed a questionnaire regarding vaccination based on the Health Belief Model (S1 Questionnaire). This questionnaire was developed for this study, but was based on constructs as described by Wong and collegeaus [17]. Due to the urgent character of the question and time constraints (due to a limited potential timeframe in which the study could take place) the questionnaire was not piloted. However, by checking correlations between items afterwards, as well as preforming latent class analysis on the items regarding barriers and benefits experienced by the target group, the structure of the questionnaire was confirmed, supporting sufficient content validity.

The questionnaire consisted out of 27 questions, including four open questions regarding opinions on vaccination, and five questions regarding background characteristics, i.e., age, gender, chronic disease status, educational background, and immigration background.

In the questionnaires the following concepts of the health belief model were described: behavior (i.e., vaccination hesitancy), perceived susceptibility, perceived severity, benefits, and barriers. Perceived susceptibility to Covid-19 infection (on a five-point scale, i.e., If you would not get vaccinated, how high would you estimate the chances of you getting Covid-19?), perceived severity of such an infection were measured on a five-point scale, i.e., If you would not get vaccinated, how severe would you estimate the symptoms would be if you would get Covid-19? using a single item. These concepts were both included as continuous predictor variables. To gain insight into the relevance of potential perceived barriers and benefits in the decision to get vaccinated several dichotomous questions were used. To gain insight into potential barriers (i.e., Did the following potential benefit of vaccination influence your decision about vaccination?) we included protection of myself, protecting my environment, helping society, contributing to opening up society, being able travel, access to facilities like restaurants of entertainment facilities, and meeting wishes of close ones, as potentials benefits. As potential barriers (i.e., Did the following potential barriers of vaccination influence your decision about covid-19 vaccination) we included, the time it takes to get vaccinated, the effort it takes to get vaccinated, the fear of side-effects, the fear of pain during vaccination, the fear of needles, objections related to religion of beliefs, and the fear of judgement of close ones. Benefits and barriers were, separately, merged in underlying constructs describing clusters of benefits and barriers, which were used as predictors. Additionally, participants answered if they had, up until the moment of vaccination, experienced hesitancy regarding vaccination (three-point scale: no hesitancy, hesitancy, rejection of vaccination). This variable was recoded into a dichotomous variable with the possible outcomes: ‘(some) vaccination hesitancy’, and ‘no vaccination hesitancy’, and was included as the outcome variable.

### Participants

We made use of a convenience sample. All people who received a vaccination at the pop-up location during the study period of four consecutive days, and were willing to participate, received a questionnaire (N = 318). However, in this study we aimed to study the decisions of young people, and therefore selected only those participants between 16 and 30 years of age (N = 194, 61%). Because the questionnaire was available in Dutch and English, only participants with enough literacy skills in one of these languages were eligible for participation. Besides just receiving a vaccination, age between 16 and 30 years of age, and being able to read and write Dutch or English, no inclusion or exclusion criteria were used in this study. Response rate was not registered, however the vast majority of eligible candidates agreed to participate.

### Analyzing procedure

Data was analyzed using IBM SPSS (version 26). Descriptive statistics were calculated for demographic background characteristics, and predictors as defined in the health belief model. The outcome variable was recoded into a dichotomous variable (no hesitancy vs. at least some hesitancy). Secondly, to prevent multicollinearity and overcomplication of the model, we looked for potential latent variables underlying the various benefits by combining these items via a latent class analysis, using a Varimax rotation (25 iterations) and a cut-off score of Eigenvalue = 1.0. A similar procedure was followed regarding barriers. Consequently, a pearson correlation analysis was conducted between background characteristics (age, gender, chronic disease status, educational background, and immigration background), predictors (perceived severity, perceived susceptibility, latent variables describing benefits, and latent variables describing barriers), and the outcome variable (vaccination hesitancy). Lastly, we included background variables that significantly correlated with the outcome measure as well as predictors as defined in the health belief model (i.e., perceived severity, perceived susceptibly, latent variables describing benefits, and latent variables describing barriers) in a binary logistic regression predicting experienced hesitancy regarding vaccination Wald Test of significance. Covariates were entered in the first block, while all other predictors were entered in the second block. All analysis effects were deemed significant if p < 0.05.

## Results

### Background characteristics

Background characteristics are depicted in Table 1. The only variable containing missing data was educational level (*n* = 7 cases missing, 3.6%). The mean age in our sample was 22.84 years, and number of male and female participants was almost equal. Most of our sample reported a high educational level (65.2%) and a Dutch background (59.3%). Chronic diseases were uncommon (9.3%), as could be expected in relatively young samples. About one third of participants (33.5%) reported that they had experienced hesitancy about vaccination, up until the moment of vaccination.

**Table 1:** Background characteristics of the complete sample (N = 194).

Characteristic	Frequency (percentage)
Sex	
Male	102 (52.6%)
Educational level*	
Low or medium	65 (34.8%)
High	122 (65.2%)
Migration background	
Yes	79 (40.7%)
Chronic disease	
Yes	18 (9.3%)
Age	
Mean, *SD*	22.84 (*4*.*96*)
Experienced hesitancy before vaccination*	
Yes, at least some hesitancy	65 (33.5%)

*Original items originally included three answering options but were recoded into dichotomous variables.

### Dimension reduction

We used a latent class analysis to identify latent variables underlying the constructs of Benefits and Barriers. The benefit of protecting others was most often seen as a relevant factor in making the decision about vaccination (88.1%), while meeting the needs of others was less often reported (36.6%). Regarding barriers, religions reasons for vaccine hesitancy were hardly reported (3.1%), while fear of side effects (38.1%) and fear of needles (23.6%) were common barriers.

When entering all benefit related items to the model, two underlying factors appeared (Eigenvalue = 2.10; Eigenvalue = 1.53), explaining 29.97% and 21.82% of variance respectively. Rotated factor loadings for benefits are depicted in Table 2, showing two clearly distinctive underlying constructs. While factor 1 describes the construct of Health related and idealistic benefits, factor 2 describes “Practical and individual benefits” of getting vaccinated.

**Table 2:** Factor loadings of benefit related items on underlying constructs with an eigenvalue of >1 as determined by latent class analysis.

Benefit related dichotomous items	Factor loadings factor 1	Factor loadings factor 2
Protecting myself	**.76**	-.13
Protecting my environment	**.70**	-.11
Helping society as a whole	**.77**	.24
Opening up society	**.53**	**.50**
Free travel	-.09	**.71**
Access to public facilities	.05	**.79**
Meeting demands of others	.02	**.46**

Factor 1 describes health related and societal benefits. Factor 2 describes practical benefits

Loadings are in bold underneath the factor they most contribute to.

A similar approach was used to identify underlying factor of Barriers. The item asking about religious concerns as a possible barrier was excluded from analysis, because only 6 participants (3.1%) indicated that this was a barrier they had considered in making their decision about vaccination. In Table 3 rotated factor loadings are depicted for the items related to barriers on two underlying factors that that were identified with latent class analysis (Eigenvalue = 2.02; Eigenvalue = 1.40), explaining 33.60% and 23.77% of variance respectively. The first factor describes a construct concerning Feelings of fear, while the second factor describes a construct concerning Practical barriers.

**Table 3:** Factor loadings of barrier related items on underlying constructs with an eigenvalue of >1 as determined by latent class analysis.

Barrier related dichotomous item	Factor loadings Factor 1	Factor loadings Factor 2
Time effort	.03	**.87**
General effort	.07	**.81**
Fear of side-effects	**.66**	-.06
Fear of pain	**.74**	.18
Fear of needles	**.75**	-.10
Fear of judgement	**.59**	.23

Factor 1 describes practical barriers. Factor 2 describes barriers related to feelings of fear

Loadings are in bold underneath the factor they most contribute to.

### Hesitancy about vaccination

#### Simple analyses

Our sample had a mean age of 22.84 (*SD = 4*.96) and was for the majority highly educated (65.2%). We first examined singular associations between the outcome variable (hesitancy about vaccination) and background characteristics as well predictors as defined by the health belief model, by calculating correlations. There was no significant association found between hesitancy and chronic disease, migration background, and sex. However, a negative association was found between experiencing hesitancy and age (*r* = -.246, *p* < .001). Participants who reported hesitancy were younger (on average 21.1 years, *SD* = 5.1), than their counterparts who were on average 23.7 (*SD* = 4.7) years of age. Because educational level was not a dichotomous or continuous variable, but was divided into three categories, we performed a chi square test. We found a significant connection between hesitancy and educational level (*χ*^*2*^ = 11.48, *p* = .003); people with low or moderate educational levels reported higher levels of hesitancy than their higher educated peers.

When correlating the constructs of the health belief model with experienced hesitancy, perceived severity did show a borderline significant association with hesitancy (*r* = -.14, *p* = .051). The association between perceived susceptibility and experienced hesitancy was significant (*r* = -.22, *p* = .003). Participants who believed infection was likely, less often reported hesitancy about vaccination. People who experienced a relatively high level of “Practical and individual benefits” of vaccination, such as being able to travel, did not show decreased levels of hesitancy (*r* = .08, *p* = .308) as compared to peers who did value these kinds of benefits. However, people who reported to have considered health related and idealistic benefits, less often reported hesitancy (*r =* -.47, *p* < .001). Also, when it comes to barriers, only one of the underlying constructs showed a significant association with reported hesitancy. While practical barriers did not seem to be related with hesitancy (*r* = -.02, *p* = .779), feelings of fear did show a significant association (*r* = .42, *p*< .001).

#### Logistic analysis

We included all background variables that showed a significant association with hesitancy about vaccination, in the logistic regression (i.e., age and educational level), as well as all constructs as described in the health belief model: perceived severity, perceived susceptibility, two types of experienced benefits (societal and practical, as defined by latent class analysis), and two types of experienced barriers (practical and feelings of fear, as defined by latent class analysis). Outcomes are presented in Table 4. Neither of the background characteristics (block 1) remained significant. Regarding all other predictors (block 2), in the logistic model, also perceived susceptibility no longer showed a significant association with reported hesitancy (*W* (1) = .16, *p =* .689). Health related and idealistic benefits still showed a significant association (*W* (1) = 23.15, *p* < .001), participants who considered these potential health benefits of vaccination were far less likely to report feelings of hesitancy. Also, barriers regarding experienced fear remained significant (*W* (1) = 20.33, *p* < .001), participants who reported high levels of fear were clearly more likely to experience hesitancy, than those who had experienced low levels of vaccination related fear and anxiety.

**Table 4:** Results of logistic regression with age, educational level and predictors as defined in the health belief model predicting hesitancy about vaccination in young adults.

Predictor	B	S.E.	Exp (B)	*P-value*	Wald statistic
Constant	.80	1.11	2.21	.472	.59
Age	-.09	.06	.91	.124	.91
Educational level	-.01	.34	.99	.983	1.02
Per. severity	-.01	.27	.99	.975	.01
Per. susceptibility	.09	.21	1.09	.689	.16
**Benefit health**	**-1.13**	**.23**	**.32**	**< .001**	**23.15**
Benefit practical	.27	.25	1.32	.280	1.11
**Barrier fear**	**1.05**	**.23**	**2.86**	**< .001**	**20.33**
Barrier practical	.11	.21	1.11	.612	.19

All predictors are measured on a continuous scale, with an exception for education, which is dichotomous.

Statistically significant results (p < .05) are printed in bold.

## Discussion

In general, among young people, COVID-19 vaccination rates are relatively low. Therefore, the current study aimed to identify barriers and motives, as defined by the health belief model, that were experienced by young people who eventually (several weeks to months after receiving the formal invitation by the government) decided to get vaccinated. The identification of these barriers and motives could offer valuable starting points for facilitating the needs of youngsters, for example by developing communication campaigns addressing the right motivations.

Young people who experience hesitancy regarding COVID-19 vaccination turned out to be younger and to have a lower educational level than young people who do not experience hesitancy. There were no associations between sex and hesitancy, and between migration background and hesitancy. Other studies find increased levels of vaccine reluctancy in ethnic minority populations [18], migration background is however often closely linked to educational level [19]. Our sample, however, included mainly highly educated participants. In this sample, migration background thus does not seem to be a relevant factor in explaining vaccination hesitancy.

Within our young sample, age and educational level were negatively correlated with vaccination doubt. This could be partly explained by the relatively high correlation between age and educational level in our sample (*r* = .635, *p <* .001), but also because both age [20] and educational level [21] have been associated with lower levels of needle anxiety. While both age and educational level were not significantly associated with the fear related barriers factor, age and educational level were negatively correlated with the items regarding fear of pain (*r* = -.197, *p* = .006; *r =* -.184, *p* = .012), educational level was also negatively associated with fear of needles (*r* = -.165, *p* = .024), supporting findings from literature. Potentially, young people experience higher levels of needle anxiety due to limited exposure to medical procedures.

Additionally, this study that young people from Rotterdam were primarily motivated to get vaccinated because of Health related and idealistic beliefs; wanting to help and protect others and society. Youth who considered this as important, experienced lower levels of hesitancy about vaccination. Besides, many young people reported that “practical and individual benefits”, like being able to travel, were important to them, but these benefits were not related to experienced hesitancy. Practical barriers, like the effort it takes to travel to a vaccination location, were not related to experienced hesitancy as well, just like perceived severity and susceptibility to COVID-19 infection. Instead, fear related barriers, such as fear for needles or side-effects, were the strongest predictor of experienced hesitancy.

Young people thus mostly seem to be altruistically motivated for vaccination, driven to help the public benefit instead of their private benefit. This willingness to help the public benefit could be partly explained by the experienced severity of the current pandemic; also, in other studies people show more willingness to help the public benefit (in this case by sharing their private health data) for fighting the COVID-pandemic than in other instances [22]. Additionally, prosocial motivations are most often reported as the main reason to adhere to COVID-19 measures by people in general, regardless of age [23, 24]. Strengthening altruistic motives, could thus be an effective way to motivate all people including young people, which is also suggested by the recent finding that in university students (mean age = 26) willingness to get vaccinated increased after triggering altruism [25].

The reasons why especially young people are motivated for vaccination by not wanting to *spread* the virus, instead of by not wanting to *get* the virus, could be explained by their relatively low risk of severe outcomes after infection. On the other hand, younger generations (generation Z, young millennials), might be expected to be used to showing behavior directed at benefitting the public benefit, such as sustainable behavior and volunteering. However, research suggests that also in young generations (i.e., millennials and generation Z), these behaviors might also be strongly driven by rational and self-oriented motives, rather dan emotional and others-oriented motives [26, 27]. Communication about the public benefits of COVID-19 vaccination, might thus benefit most from not only using emotional appeals, but from using also rational arguments and highlighting long-term benefits for society.

While young people’s motivation for COVID-19 vaccination seem to be mostly related to public benefits, experienced barriers seemed to be primarily related to fear for personal consequences, primarily related to health and the medical procedure. Respondents reported high levels of needle anxiety and fear of pain during the procedure, which is in line with the established association between a younger age and needle anxiety [28], which could partly explain why in the current study, younger participants more often reported hesitancy about vaccination. To facilitate vaccination in young people, considering this fear of needles by normalizing it and offering solutions such as having vaccinations done by especially trained vaccine workers or offering anesthetic cream before the procedure [29, 30] should be considered. In addition to fear of needles and pain during the procedure, participants reported fear for experiencing severe side-effects. Although this study did not specify which side-effects participants feared, young people might be especially concerned about potential consequences for fertility or pregnancy [31]. Providing young people with additional information on the effects of vaccination on fertility and pregnancy could potentially lift these barriers.

### Strengths and limitations

The strengths of this study are that it addresses an important topic and offers a theory-based overview of experienced motives and barriers for COVID-19 vaccination within a large sample of young people. Gaining more information about this group is especially important, because their vaccination rate is relatively low. Therefore, this study offers specific and practical action perspectives for policy and communication professionals who aim to reach and motivate this group.

However, this study, unavoidably, also knows several limitations. Our study sample mainly consisted out of highly educated participants. Motives and barriers might be different amongst young people with a low educational level. Although a substantial proportion of participants reported to have experienced hesitancy, we only consulted participants who eventually decided to get a COVID-19 vaccination, thereby not reaching those most hesitant about vaccination. Additionally, this study used self-report, which can be susceptible to bias and faults in memory. Lastly, only data of participants willing to participate were collected, however almost all eligible participants were willing to fill out the questionnaire. People not proficient in the Dutch or English written language, were however not eligible.

Additionally, barriers and benefits identified in the current study might be only applicable to young people living in Europe or other parts of the western world. A study among young people in Saudi Arabia for example identified other reasons for high levels of vaccination hesitancy, namely doubts about the safety and efficacy of the Covid-19 vaccination [12].

### Implications for future research

Future research should aim to understand barriers experienced by groups of young people who did not get vaccinated. Reasons could be skepticism, believing in conspiracy theories, and idealistic arguments against vaccinations, but could also lie in strategical or practical considerations: the relatively high infection and recovery rates in this population (based on national registration of infection rates; coronadashboard.nl) might for example result in many young people currently believing to be immune and thus postponing vaccination instead of rejecting it. Because the current sample consisted mainly of highly educated participants, and we see that lower educated groups experience higher levels of hesitancy, future studies should also focus on reaching young groups with lower educational levels, to explore their attitudes and needs.

## Conclusions

We studied motivations to get vaccinated against COVID-19 in a group of young people (16–30 years of age) who did not respond to their formal invitation for vaccination, but eventually decided to get vaccinated at a pop-up vaccination location. Our study shows that these young people were mainly motivated to get vaccinated by idealistic and health related benefits, while practical and individual benefits (such as being able to travel) were not associated with experienced hesitancy. On the other hand, practical barriers did not explain hesitancy, while anxiety related barriers were a strong predictor for vaccination hesitancy. Policy makers and communication experts can use current results when trying to reach and motivate young people to get vaccinated against Covid-19. Motivating young people by emphasizing health related and idealistic benefits of vaccination, might be an effective approach. Additionally, lifting barriers related to anxiety could be a promising strategy to facilitate vaccination, for example by offering additional support during vaccination.

## Supporting information

S1 Questionnaire Questionnaire as handed out to the participants, English version. (DOCX)
