# Supplementary material for: Using the health belief model to explain COVID-19 vaccination hesitancy in Dutch urban citizens under thirty
Source: PLoS One. 2023 Jan 26;18(1):e0279453. doi: 10.1371/journal.pone.0279453 (PMC9879493; doi:10.1371/journal.pone.0279453)
Supplement: S1 Questionnaire — (DOCX) [file pone.0279453.s001.docx]

**Questionnaire Vaccination at the Maassilo**

1. **Why did you decide to get a vaccination?**
2. **Why did you decide to get a vaccination *here* and *now*?**
3. **Before today, did you already intend to get vaccinated?**
    Yes
    No
    I was in doubt
4. **Did conversations with or messages from the following individuals or organizations have impact on your decision about getting a vaccination?**

|  | Yes, in a manner positive towards vaccination | Yes, in a manner negative towards vaccination | No |
| --- | --- | --- | --- |
| The government | ⃝ | ⃝ | ⃝ |
| Physician(s) | ⃝ | ⃝ | ⃝ |
| Family members | ⃝ | ⃝ | ⃝ |
| Friends | ⃝ | ⃝ | ⃝ |

1. **If you would not get vaccinated, how likely do you think it would be for you to get Covid-19?**

 Very unlikely

 Unlikely

 Somewhat likely

 Likely

 Very likely

1. **Did this influence your decision to get vaccinated?**

 Yes

 No

1. **How serious do you think the consequences for you would be if you were to get COVID-19?**

 Not serious at all

 Not serious

 Somewhat serious

 Serious

 Very serious

1. **Did this influence your decision to get vaccinated?**

 Yes

 No

1. **Did the following advantages of vaccination play a role in your decision to get vaccinated?**

|  | Yes | No |
| --- | --- | --- |
| Protecting myself against Covid-19 | ⃝ | ⃝ |
| Protecting people around me | ⃝ | ⃝ |
| Regaining entry to facilities or events | ⃝ | ⃝ |
| Opening up society as a whole | ⃝ | ⃝ |
| Traveling freely | ⃝ | ⃝ |
| Adhering to the wishes of people around me | ⃝ | ⃝ |
| Helping society as a whole | ⃝ | ⃝ |

1. **Did the following disadvantages play a role inyour decision to get vaccinated?**

|  | Yes | No |
| --- | --- | --- |
| Pain during vaccination | ⃝ | ⃝ |
| Fear of potential (serious) side effects | ⃝ | ⃝ |
| The time it takes to get vaccinated | ⃝ | ⃝ |
| The difficulty it takes to get vaccinated (e.g., difficult to reach vaccination site) | ⃝ | ⃝ |
| Fear of needles or injections | ⃝ | ⃝ |
| Religious objections or beliefs that are not in line with vaccination | ⃝ | ⃝ |
| Disapproval from people around me | ⃝ | ⃝ |

1. **Do you have confidence in the Dutch health care system?**

 Yes

 No

1. **Did this influence your decision about vaccination?**

 Yes

 No

1. **Do you have confidence in your general practitioner (GP)?**

 Yes

 No

1. **Did this influence your decision about vaccination?**

 Yes

 No

1. **Do you have confidence in companies that manufacture vaccines?**

 Yes

 No

1. **Did this influence your decision about vaccination?**

 Yes

 No

1. **Do you agree with the following statements about vaccines and vaccination?**

|  | Agree | Disagree |
| --- | --- | --- |
| The vaccine protects me | ⃝ | ⃝ |
| The vaccine protects my environment (because I’m less contagious for other people) | ⃝ | ⃝ |
| I’m afraid of side effects of the vaccine | ⃝ | ⃝ |
| The vaccine has been tested sufficiently | ⃝ | ⃝ |
| I find the information about vaccination clear | ⃝ | ⃝ |
| After taking the vaccine, I cannot get infected with Covid-19 anymore | ⃝ | ⃝ |
| People who have already had Covid-19 still need to get vaccinated | ⃝ | ⃝ |

1. **What is your age?**
2. **What is your sex?**
3. **What is your highest completed education?**
4. **Whith which cultural or ethnic background do you identify?**
5. **Do you have a chronic disease? If so, which one(s)?**
